# Molecular Profile as an Outcome Predictor in Glioblastoma along with MRI Features and Surgical Resection: A Scoping Review

**DOI:** 10.3390/ijms25179714

**Published:** 2024-09-08

**Authors:** Serban Iancu Papacocea, Daniela Vrinceanu, Mihai Dumitru, Felicia Manole, Crenguta Serboiu, Marius Toma Papacocea

**Affiliations:** 1 Neurosurgery Department, Carol Davila University of Medicine and Pharmacy, 020021 Bucharest, Romania; serban-iancu.papacocea@drd.umfcd.ro (S.I.P.); toma.papacocea@umfcd.ro (M.T.P.); 2 ENT Department, Carol Davila University of Medicine and Pharmacy, 020021 Bucharest, Romania; orldumitrumihai@yahoo.com; 3 ENT Department, Faculty of Medicine, University of Oradea, 410073 Oradea, Romania; manole.felicia@gmail.com; 4 Cellular Biology and Histology Department, Carol Davila University of Medicine and Pharmacy, 020021 Bucharest, Romania; crengutas@yahoo.com

**Keywords:** GBM, molecular, predictor, outcome, surgery

## Abstract

Glioblastoma (GBM) is one of the most aggressive malignant tumors of the brain. We queried PubMed for articles about molecular predictor markers in GBM. This scoping review aims to analyze the most important outcome predictors in patients with GBM and to compare these factors in terms of absolute months of survival benefit and percentages. Performing a gross total resection for patients with GBM undergoing optimal chemo- and radiotherapy provides a significant benefit in overall survival compared to those patients who received a subtotal or partial resection. However, compared to *IDH-Wildtype* GBMs, patients with *IDH-Mutant 1/2* GBMs have an increased survival. MGMT promoter methylation status is another strong outcome predictor for patients with GBM. In the reviewed literature, patients with methylated MGMT promoter lived approximately 50% to 90% longer than those with an unmethylated MGMT gene promoter. Moreover, KPS is an important predictor of survival and quality of life, demonstrating that we should refrain from aggressive surgery in important brain areas. As new therapies (such as TTFs) emerge, we are optimistic that the overall median survival will increase, even for *IDH-Wildtype* GBMs. In conclusion, molecular profiles are stronger outcome predictors than the extent of neurosurgical resection for GBM.

## 1. Introduction

Glioblastoma (GBM) represents the most aggressive and the most frequent primary malignant tumor of the brain. Its diffuse and infiltrative nature usually correlates with a poor prognosis and a mean rate of survival of approximately 12–15 months, in spite of recent medical and technological advances in the standard of care [1,2,3]. Nevertheless, recent developments in genetic and molecular studies have allowed for a better understanding of the pathology of GBM, as well as offering a more realistic view upon classification and clinical outcomes. In this review, we aim to analyze the most important outcome predictors in patients with GBM, to quantify their impact, and to compare these factors to each other both in terms of absolute months of survival benefit and percentages.

According to the American Association of Neurosurgical Societies (AANS), the incidence of GBM is about 3.2 per 100,000 patients, with a median age of 64 years [4,5]. Globally, the incidence is similar, ranging from 3 to 4 per 100,000 people. Since the median survival time in patients with GBM is close to one year, the global prevalence is difficult to estimate. It is considered to be similar to the number of newly diagnosed cases per year, which is approximately 250,000–300,000 globally [1]. In the adult population, GBM accounts for about 14.5% of all CNS tumors (excluding meningiomas) and for approximately half (48.6–49%) of all malignant primary brain tumors, making it the most common primary malignancy of the brain [6,7]. GBM is also the deadliest form of cancer, having a mean 5-year survival rate of only 5% and a 10-year survival of less than 1% [8]. The literature is extremely scarce regarding the global mortality rate of GBM patients. However, some studies estimate a 1-year mortality of 60% and a 2-year mortality of 83% [1]. That is equivalent to approximately 200,000–250,000 yearly deaths by GBM worldwide.

Although GBMs were presumed to arise from astrocytes, more recent studies are controversial, some suggesting that GBM arises from neural stem cells from the SVZ (Subventricular Zone), while others suggest that the occurrence of GBM is a result of astrocytic de-differentiation [9,10,11,12].

Classically, GBMs were divided into primary and secondary types. According to the WHO 2016 classifications of diffuse gliomas, primary GBM referred to malignant tumors arising de novo, usually in older patients, whereas secondary GBM referred to WHO grade 2 or 3 gliomas undergoing further malignization, more often in younger patients. According to this classification, primary GBMs represent 90% of all GBMs, whereas secondary GBMs account for 10% [10,13,14]. There are certain molecular, genetic, and outcome factors depending on this classification. For example, secondary GBM is associated with *TP53* and *ATRX* mutations; more often than not, they are *IDH-Mutant 1/2*, whereas primary GBM is usually *IDH-Wildtype* [15,16,17]. Today, WHO 2016 is considered outdated. WHO 2021, which is the currently accepted classification for tumors of the nervous system, has eliminated the concept of secondary or IDH-Mutant 1/2 GBM. According to this classification, GBMs are by definition primary and *IDH-Wildtype*. Tumors that were previously considered “IDH-mutant, secondary GBMs”, though histologically similar to primary GBMs, should be considered separate pathologies, as they are now defined as grade 4 astrocytomas [18]. This further emphasizes the idea that genetic and molecular differences might be a stronger predictor of outcome than histopathological characteristics or even than the extent of resection, a hypothesis that we will review in the following section. Although many of the articles reviewed in the following section were written before 2021, following the WHO 2016 classification and using terms such as “primary” and “secondary” glioblastoma, we will adapt to the WHO 2021 classification and use the term “grade 4 astrocytoma” whenever the term “secondary or IDH-mutant glioblastoma” is mentioned in the literature. We will use the term “grade 4 gliomas” to include both grade 4 astrocytomas (referred to as secondary GBMs in older studies) and GBMs (this category includes the term “primary GBMs” from older studies).

## 2. Material and Method

**Figure 1:**
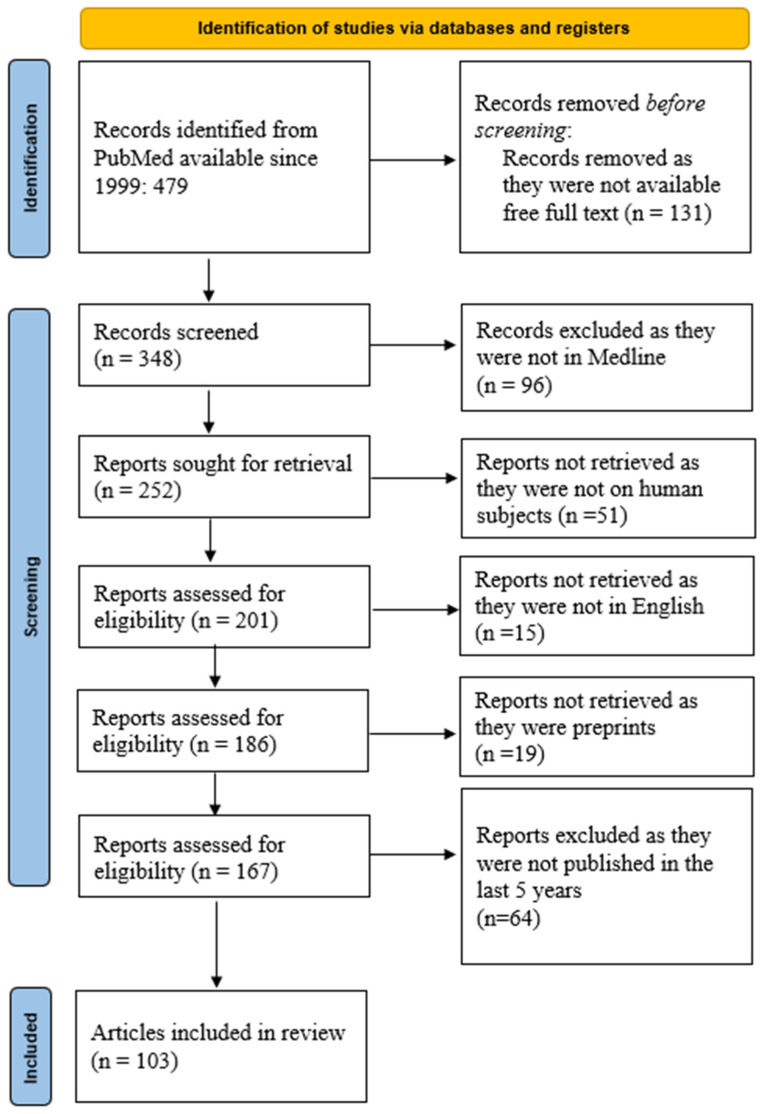
PRISMA diagram of the current scoping review.

We queried the PubMed database using the following keywords: molecular profile prediction GBM. We obtained 479 manuscripts from the last 25 years. Further restriction of the research to articles where the free full text was available limits the number of manuscripts to 348. In the Medline database, there are 252 articles. From these, there are 201 manuscripts on human subjects, of which 186 are written in English. We also exclude preprints and obtain 167 articles. Further limiting the research to the last 5 years, we obtain 103 articles, as shown in Figure 1. The search syntax was as follows: ((“molecular”[All Fields] OR “moleculars”[All Fields]) AND (“profile”[All Fields] OR “profiled”[All Fields] OR “profiler”[All Fields] OR “profilers”[All Fields] OR “profiles”[All Fields] OR “profiling”[All Fields] OR “profilings”[All Fields]) AND (“predict”[All Fields] OR “predictabilities”[All Fields] OR “predictability”[All Fields] OR “predictable”[All Fields] OR “predictably”[All Fields] OR “predicted”[All Fields] OR “predicting”[All Fields] OR “prediction”[All Fields] OR “predictions”[All Fields] OR “predictive”[All Fields] OR “predictively”[All Fields] OR “predictiveness”[All Fields] OR “predictives”[All Fields] OR “predictivities”[All Fields] OR “predictivity”[All Fields] OR “predicts”[All Fields]) AND (“glioblastoma”[MeSH Terms] OR “glioblastoma”[All Fields] OR “glioblastomas”[All Fields])) AND ((y_5[Filter]) AND (ffrft[Filter]) AND (excludepreprints[Filter] OR medline[Filter]) AND (humans[Filter]) AND (english[Filter])).

The steps followed in designing this scoping review were as follows: (1) we identified the research question, (2) we identified relevant studies, (3) we selected studies using an iterative team approach, (4) we charted the data incorporating numerical summary and qualitative thematic analysis, and (5) we summarized and reported the results.

PubMed search results outlined in Figure 1 were imported into one online cloud database. Subsequently, two groups of two reviewers (S.P. and D.V.; M.D. and M.T.P.) screened titles, abstracts, and full texts for inclusion, independently. All discrepancies between reviewers were resolved by another set of two reviewers (F.M. and C.S.). This type of reviewer distribution and control is one of the solutions to limit possible bias.

## 3. Results

Although several advancements have been made in classifying and treating GBM, it is widely accepted that, due to its diffuse and infiltrative nature, this tumor is virtually incurable and that survival times usually fall below 2 years, even with the best existing medical and surgical treatment. However, there are certain intrinsic factors of GBMs that are associated with a better or worse prognosis, regardless of treatment.

### 3.1. IDH-Mutant 1/2 Grade 4 Astrocytoma (Previously Known as Secondary GBM) vs. GBM (IDH-Wildtype)

It is currently widely accepted that IDH-Mutant 1/2 grade 4 astrocytomas have a better outcome than their *IDH-Wildtype* counterparts. However, this fact has been observed in studies as old as 20 years—even before the discovery of the IDH mutations in GBM. For example, a 2001 study showed that the absence of the *TP53* mutation is a predictor of poor outcome and lower survival rates [19]. Today, we know that *TP53* is associated with IDH-Mutant gliomas, whereas the absence of the *TP53* mutation is associated with GBM (*IDH-Wildtype*). These findings are further backed by a 2004 study which analyzed a population of 1 million Swiss residents, from 1980 to 1994, diagnosing approximately 700 patients with GBM [20]. Those patients were treated with surgery and radiotherapy. This study shows that, even in the pre-temozolomide era, grade 4 astrocytomas, associated with the *TP53 mutation,* have a better outcome (median survival of 7.8 months) compared to GBMs (median survival of 4.7 months). In other words, even in populations analyzed 7 years before the introduction of temozolomide as a second-line drug for GBM in 1999 and as a first-line agent later on and 14 years before the discovery of the importance of the *IDH mutation* in GBM patients in 2008, some studies were able to establish a strong correlation between the genetic markers of grade 4 gliomas and survival outcome [21,22,23]. At that time, the involvement of IDH mutation in GBM survival had not been discovered. The population involved in this study [20] was analyzed before the introduction of temozolomide as a main chemotherapeutic drug for the treatment of GBM. As such, a comparison is made between the absence of the TP53 mutation and GBM (mentioned as primary GBM in the study) on the one side and the presence of the TP53 mutation and grade 4 astrocytoma (mentioned as secondary GBM in the study) on the other side. It is reasonable to assume that the former group were overwhelmingly *IDH-Wildtype*, whereas the latter were, most probably, IDH-Mutant. Today, we know that GBM is *IDH-Wildtype*, whereas grade 4 astrocytoma is usually *IDH-Mutant 1/2* and carries a better prognosis. However, this is not always the case. For example, IDH-Mutant tumors exhibiting CDKN2A mutations were associated with aggressive behavior and a mean survival time of 1.6 years, comparable to GBM, whereas IDH-Mutant tumors without this mutation have a mean survival time of 12.6 years [24]. It is worth noticing that, following the introduction of temozolomide, in terms of percentages, the difference in median survival between GBM and grade 4 astrocytoma (referred to in older studies as secondary glioblastoma) has improved as well. In order to demonstrate this claim, we will compare the differences from the pre-temozolomide era to the median survival time obtained using current protocols. Temozolomide is an alkylating agent that leads to apoptosis by blocking the cell cycle at G2/M [25]. As previously shown, before the approval of temozolomide in 1999, the median survival times for GBM were about 4.7 months, compared to 7.8 months for grade 4 astrocytoma [20]. Though a difference of 3.1 months in survival may not seem significant, it represents 66% of the GBM population median survival time.

A large study in 2005 showed that patients treated with surgery and both temozolomide and radiotherapy have a median survival time 2.5 months longer than patients treated with surgery and radiotherapy alone, at 14.6 months [26]. Two decades later, this is still the standard of care for GBM, consisting of aggressive surgery (gross total resection) associated with radiotherapy and temozolomide, known as the Stupp protocol.

For patients with GBM (*IDH-Wildtype*) receiving the standard protocol, a Chinese study found a median survival time of 14.6 months [27]. Other studies place the median survival time for GBM between 10 and 15 months [28,29,30,31]. Conversely, the median survival time for *IDH-Mutant 1/2* grade 4 astrocytoma was 18.6 months from the moment of conversion (from a grade 2 or 3 astrocytoma GBM) [32].

Data extracted from the Kaplan–Meyer plots of a 2010 study also show a median survival time of 12 months for GBM (*IDH-Wildtype*) and a 36-month median survival time for *IDH-Mutant 1/2* grade 4 astrocytoma [33]. Lastly, a study published in NEJM showed a median overall survival of 15 and 31 months for *IDH-Wildtype* and *IDH-Mutant,* respectively [34]. Some of the comparisons are summarized in Table 1.

As can be observed, since the introduction of temozolomide as the main chemotherapeutic drug for treating grade 4 gliomas, the overall survival time has not improved significantly in matters of absolute time, especially for *IDH-Wildtype* patients. However, what has changed is the gap in survival between *IDH-Wildtype* and *IDH-Mutant 1/2* patients. If patients that were only treated with surgery and radiotherapy survived 66% longer in case of *IDH-Mutant 1/2* grade 4 glioma compared to its *IDH-Wildtype* counterpart, patients with *IDH-mutant 1/2 grade 4 astrocytomas* treated with temozolomide (and surgery + radiotherapy) live 100% or even 200% longer than *IDH-Wildtype GBM* patients treated with the exact same protocol in some studies [33]. The logical conclusion would be that *IDH-Mutant grade 4 gliomas* respond better to temozolomide than the *IDH-Wildtype* ones. This claim is backed by several studies, which is consistent with our conclusion [34,35,36].

### 3.2. MGMT Promoter Methylation Status

MGMT (O-6-Methylguanine-DNA Methyltransferase) is a DNA-repairing enzyme that protects against the action of alkylating agents. It is encoded by the MGMT gene. Methylation of this gene’s promoter leads to a decreased synthesis of the MGMT enzyme and has been independently associated with increased survival in infiltrating gliomas, including GBMs, as a result of an increased response to temozolomide [36,37,38,39]. Reciprocally, unmethylated MGMT promoter status leads to an abundance of the MGMT enzyme and is associated with poor response to temozolomide and a decreased survival in patients with GBM and other infiltrating gliomas [40]. For a better understanding of this phenomenon, it is imperative that we elaborate on the mechanisms of action of temozolomide and MGMT [41].

Temozolomide is an alkylating agent that attaches a methyl group to DNA purine bases (adenine or guanine). The methylation can either take place at the N-3 position of adenine or at the N-7 or O-6 positions guanine [42]. Since the latter is considered the essential step in inducing cell death [42,43], we will solely elaborate on the methylation of the O-6 guanine residue. Methylated guanine will bind with thymine instead of cytosine. This mispair is detected by the MMR (DNA Mismatch Repair) system, which removes the thymine residue from the strand. However, since the guanine residue is still methylated, it will be paired with thymine once again. These repeated cut-and-reattach sequences will eventually lead to DNA damage and tumor cell apoptosis [44,45,46].

The main function of MGMT is to remove the anomalous methyl group from the guanine residue [47]. This will allow the guanine residue to bind with cytosine once again and undergo cell division normally. As such, cells that exhibit a high activity of the MGMT promoter will have an abundance of MGMT, thus canceling the cytotoxic effect of temozolomide, by undoing the methylation of the guanine residue. This is one of the most well-known mechanisms of temozolomide resistance in infiltrative gliomas, including GBMs [44]. Conversely, hypermethylation of the MGMT promoter will lead to a low activity of the MGMT enzyme, decreasing the cells’ ability to repair their DNA, making them susceptible to temozolomide and other cytotoxic alkylating agents. It is also important to underline the role of the MMR system. Deficiencies in this system will cause the abnormal pair of methylated guanine and thymine (MeG = T) to go unnoticed. Therefore, the tumor cell will still undergo division, even with this mismatch, rendering temozolomide useless. In other words, even in the absence or low expression of MGMT, a deficiency in the MMR system will induce temozolomide resistance [45].

Having tumor cells which are more susceptible to cytotoxic chemotherapy naturally increases the median survival time. In the next part of this section, we will try to quantify the effect of MGMT activity on median survival.

The benefit of having a low MGMT expression has been proven for almost 20 years. A 2005 study published in the New England Journal of Medicine demonstrated a median survival of 18.2 months for those patients with a hypermethylated MGMT promoter (equivalent to a lower MGMT enzyme activity) and a median survival of 12.2 months for those with an active MGMT promoter (equivalent to an abundance of the MGMT enzyme), which represents a survival benefit of approximately 50% [37]. These data are supported by a large randomized trial—the DIRECTOR trial—published 10 years later, in 2015, which showed that the unmethylated status of the MGMT promoter is associated with a median survival of 7.9 months, compared to 12.5 months for the methylated MGMT promoter. In terms of percentages, this is equivalent to a survival benefit of about 63% for the latter. This study has also showed that the survival rate 12 months after the first temozolomide administration was 23% for the unmethylated MGMT promoter and 54% for the methylated one [38]. Additionally, one study published in 2014 showed that the *IDH1 Mutation* together with MGMT promoter status are a better survival predictor than either *IDH 1* or MGMT status taken separately [39] (Table 2).

### 3.3. Alterations of the Telomerase Reverse Transcriptase (TERT) Promoter

Telomeres represent repetitive nucleotide sequences at the end of linear chromosomes. The repeated sequence is TTAGGG [48]. After each cell cycle, a part of these nucleotides is “consumed”, therefore shortening the telomere [49]. Telomerase is a ribonucleoprotein enzyme complex that consists of two components: the reverse transcriptase—human TERT gene (hTERT)—and an RNA component (TERC). The enzymatic complex adds repeated TTAGGG nucleotide sequences at the end of the chromosome, thus repairing telomeres and prolonging cell life [50]. In normal cells, telomerase has a relatively low activity as a result of TERT gene silencing. As such, the telomeres keep shortening, eventually leading to cell aging and apoptosis. TERT silencing is considered one of the most important tumor-suppressing factors in humans [51,52,53,54]. Approximately 90% of human tumor cells have an upregulation of TERT activity. These cells will not undergo normal apoptosis; instead, increased TERT activity leads to so-called “cell immortality” and cancer progression [53]. In vitro studies performed on various human cells have shown that 98% of the immortal cells and 0% of the non-immortal cells present TERT activity [55].

In GBM, two TERT promoter mutations have been described—C228T and C250. A 2013 study analyzed a group of 358 cases of grade 4 gliomas, of which 322 were GBM (*IDH-Wildtype*) and 36 were IDH-Mutant grade 4 astrocytoma. This study showed that TERT promoter mutation is associated with *IDH-Wildtype* in 58% of cases and with IDH Mutation 1/2 in only 28% of cases [56]. A more recent study showed that GBM (*IDH-Wildtype*) is associated with TERT promoter mutations in 89% of the cases, compared to only 20% in IDH-Mutant astrocytomas. The reason why some studies [57,58] consider TERT promoter mutations as negative prognostic factors of survival is because of their strong correlation with *IDH-Wildtype* status and the strong inverse correlation with IDH-Mutant status. However, multivariate analyses have shown that TERT does not represent an independent prognostic factor when IDH-Mutant and Wildtype grade 4 gliomas are analyzed separately [56,59]. The comparison of survival data between GBM patients with and without mutations of the TERT promoter is underlined in Table 3.

### 3.4. EGFR Mutations in Glioblastoma

EGFR is a transmembrane glycoprotein belonging to the receptor tyrosine kinase family [60]. EGFR alterations have been described in several other malignancies such as colon cancer [61] and non-small-cell lung cancer (NSCLC) [62]. In glioblastoma, both EGFR gene amplification and mutations have been described. EGFR gene amplification is the most frequent genetic alteration in glioblastoma, leading to an overexpression of EGFR that occurs in approximately a half of GBMs [63]. The most frequent mutation is variant III (EGFRvIII); both overexpression and EGFRvIII have been associated with increased invasiveness [60,64,65].

Montano N. et al. (2011) showed that GBMs associated with EGFRvIII, MGMT hypermethylation, and a Ki67 index of less than 20% lead to an increased overall survival. However, since the latter two represent beneficial survival factors of their own, it is unclear whether the EGFRvIII mutation offers a clear benefit in survival. Nevertheless, the same study showed that EGFRvIII GBM cells have an increased response to temozolomide in vitro [63].

An older study from 2003 has shown that EGFR overexpression is a negative prognostic factor, with a median survival time of 1.2 years, compared to 1.7 years for those without EGFR overexpression, while EGFRvIII did not have a significant impact on survival [66]. More recent studies [67,68] have shown that neither EGFR overexpression nor the EGFRvIII mutation have a significant impact on survival. Lastly, one study from 2012 has obtained paradoxical results, showing that patients with high or absent amplification had a good response to temozolomide, having a twice as long a survival time compared to patients with same EGFR status that did not receive temozolomide. On the other hand, patients with low-to-moderate EGFR amplification had a similar outcome, whether they received temozolomide or not. In other words, patients with lower levels of EGFR amplification had a worse response to temozolomide compared to those with high EGFR amplification and to those with no EGFR amplification. As such, low-to-moderate EGFR amplification represents a negative outcome factor, whilst high or absent amplification have no impact on overall survival [69]. These findings show us that although EGFR alterations are fundamental in GBMs, their predictive value is controversial.

As the molecular understanding of cancers has improved, several therapies targeting EGFR in cancers have been developed. However, unlike NSCLC or colorectal cancer, GBM remains virtually irresponsive to EGFR-targeting therapies [70,71]. Table 4 summarizes the differences in median survival taking into account the EGFR marker.

## 4. Discussion

In this section we will focus on other predictors of the prognosis of GBM and critically compare these predictors with the efficiency of the molecular prognostic markers.

### 4.1. Clinical Aspects

Infiltrative brain tumors are highly debilitating pathologies [72]. Malignant brain tumors generate approximately 8.7 million Disability-Adjusted Life Years (DALYs) [73], being the second cause of cancer-related disability in adolescents and young adults [74].

In 1948, Dr. David Karnofsky elaborated a scoring system that assessed the independence and functionality of cancer patients. It has a maximum of 100 points, which is the equivalent of being asymptomatic, decreasing by 10 points on each level of functionality, with a score of 10 meaning the patient is moribund and 0 equating death [75]. Even 75 years later, it is still one of the most used scales for assessing the prognosis and quality of life of oncologic patients, and it is especially useful for GBM patients, because of the debilitating nature of infiltrative brain tumors.

The Karnofsky performance score (KPS) correlates positively with median survival time in most literature studies. A recent study performed on a cohort from Beijing demonstrated that patients that survive more than 5 years after being diagnosed with GBM exhibit a mean Karnofsky score of 82.2 and a mean age of 41.2. By contrast, those who survived less than 1 year had a mean Karnofsky score of 76.2 and a mean age of 49.9. Naturally, this is also correlated with the *IDH-Mutant 1/2* status, which was a confirmed marker of long-term (over 5 years) survival in the same study [76]. Several other studies are consistent with these results. A retrospective cohort on elderly patients has shown that patients treated with surgery and temozolomide had significantly increased overall survival if they had a KPS of 70 or higher (8.7 months) compared to those with a KPS of 60 or lower (4.9 months). It is worth mentioning that patients in the former group had a higher time to tumor progression (TTP) than those in the latter group (5.1 months vs. 2.9 months) [77]. Lastly, one study from 2018 showed that every 10 points lost on the preoperative KPS were equivalent to an increase in mortality of 5.2%, whereas every 10 points lost on the postoperative KPS were equivalent to an increase in mortality of 4.6% [78].

### 4.2. Anatomical Aspects and MRI Features

As shown before, it has been hypothesized that GBM can originate from subventricular/subependymal stem cells [12]. Thus, the anatomical relationship of the tumor with the lateral and third ventricle has been taken into consideration as a possible predictor of survival, though the results are controversial. Some studies suggest that periventricular location can represent a negative predictor of survival [79], while other authors concluded that there is no significant difference in outcome based on distance from the tumor center to the ventricular zoneThe same study contradicts the aforementioned hypothesis, stating that only half of GBMs are in contact with the ventricular wall preoperatively, while almost 90% were located in the vicinity of the cortical area, also stating that preoperative tumor volume is neither correlated with overall survival nor with recurrence volume [80].

Though the anatomic relation with the ventricular walls is inconclusive, the location of the tumor can definitely impact prognosis. Since GBM can arise in any lobe of the cerebral hemispheres, that means it can also arise in eloquent areas. This fact has two main consequences. Firstly, a tumor arising in an eloquent area, such as the speech, motor, or visual cortex will obviously be associated with a decrease in the Karnofsky performance scale, which has been proven to be an independent predictor of mortality. It has been demonstrated that motor and verbal deficits as a result of damage to the respective eloquent areas are associated with a reduced overall survival [81]. Secondly, neurosurgeons strive to spare eloquent brain areas, aiming for the “Maximum Safe Resection”, therefore rendering GTR (gross total resection) unachievable for tumors arising in eloquent brain areas [82].

As far as imaging is concerned, there are several tests that can be performed in order to characterize and assess a GBM. One of the most used and reliable imaging tests is the contrast-enhanced MRI [83]. Although GBMs are very heterogenous tumors, both histologically and on imaging studies, there are several MRI characteristics which, when observed together, are highly suggestive for GBM, as follows:

(a) Non-enhancing central area of necrosis: Intratumoral necrosis is usually a marker of aggressive and fast-growing tumors. As the tumor cell population increases and the tumor expands, the oxygen demand increases as well [84]. GBM is also characterized by microvascular proliferation. However, these vessels are often disorganized, structurally abnormal and, therefore, inefficient in providing an adequate blood supply [85]. In other words, the angiogenesis does not “keep up” with the tumor expansion. Lastly, tumor cells, especially those in GBM, have impaired or altered apoptosis mechanisms. As a result of these circumstances, cells at the center of the tumor suffer necrosis as a result of hypoxia and nutrient deprivation [85,86].
(b) Peripheral ring enhancement: An essential imaging element of GBM, it can be used to assess the tumor both preoperatively and postoperatively. The aspect of the postoperative residual enhancement is used to quantify and define the extent of resection. However, it is worth noting that the absence of enhancement does not equate to the absence of tumor cells [87].
(c) Peritumoral edema: Unlike the peritumoral edema characterizing meningiomas or metastases, which is pure vasogenic edema, in the case of GBM, the edema is tumor-infiltrated. These two types can be distinguished from each other on MRI using axial diffusivity and radial diffusivity [88].

As far as imaging is concerned, there are several MRI findings that occur frequently in glioblastoma and in other brain tumors: pseudoprogression and pseudoresponse. Pseudoprogression is a phenomenon that refers to an apparent tumor enlargement on imaging studies, as a result of increased vascular permeability caused by temozolomide treatment which leads to increased contrast enhancement and vasogenic edema [89]. Real tumor progression implies, by definition, one of the following: increase in tumor size, tumor present in new areas, and clear neurological deterioration [90]. A cohort study of malignant glioma patients showed that out of 68 patients with GBM that were treated with temozolomide, 31 had early progression, of which 15 were considered pseudoprogressions. Therefore, approximately 22% of all GBM patients in this study showed signs of pseudoprogression [91]. Intuitively, pseudoprogressions occur more often in patients with MGMT hypermethylation [89,92], since these GBMs are more responsive to temozolomide, as shown in the previous sections. Distinguishing between real tumor progression and pseudoprogression may seem a challenging task. Assuming there are no new neurological deficits and no new tumor localizations, there is no reliable way to distinguish between them on classic MRI studies (T1 and T2) [93]. However, more advanced MRI techniques, such as the ADC (apparent diffusion coefficient) can be useful in distinguishing between real and pseudoprogression. As such, tumors that respond to treatment will have increased levels of ADC as a result of cell death [94] compared to tumors that do not respond to chemotherapy.

On the other hand, pseudoresponse represents an apparent reduction in tumor size on MRI scans after treatment with anti-angiogenic drugs. Although these chemotherapeutics are not a part of the standard protocol in GBM, one VEGF inhibitor, namely Bevacizumab, a monoclonal antibody, has been approved for the treatment of recurrent GBM after several clinical trials proving its benefit [95,96]. Inhibitors of angiogenesis decrease vascular permeability by stabilizing the brain–blood barrier, therefore reducing edema, mass effect, and enhancement [89]. On imaging studies, this will appear as a reduction in tumor size and edema, although from a cellular point of view, there is no tumor reduction. Like in the case of pseudoprogression, ADC sequences are useful to distinguish between real and pseudoresponse. Real tumor response is characterized by tumor cell death which leads to increased ADC values, whereas in pseudoresponse, the ADC levels remain low [97].

In the following section, we will analyze the impact of surgery or, more specifically, the impact of the extent of resection on the median survival of patients with GBM.

### 4.3. Extent of Surgical Resection

In GBM, there is no clear intraoperative delineation between the tumor and normal tissue, and malignant cells usually infiltrate white matter far beyond the apparent margins of the tumor and far beyond the ring enhancement on MRI studies. As such, surgery as a single treatment is obsolete. However, the extent of surgical resection is essential for patient outcome, as we shall demonstrate in the following section.

In order to fully understand the impact of surgical resection on patient prognosis, we must establish objective definitions, as follows:

- Gross total resection (GTR) is equivalent to the absence of enhancement on postoperative MRI.
- Near total resection (NTR) is equivalent to the existence of a rim enhancement on the resection cavity on postoperative MRI.
- Subtotal resection/partial resection (STR) implies the existence of a nodular enhancement on postoperative MRI.
- Partial resection is a resection of less than 95% of the tumor, based on contrast-enhanced MRI [98].
- Supramaximal resection (SMR) is the absence of any enhancement on postoperative MRI plus extending the resection into apparently normal brain tissue. Advocates of this concept argue that malignant cells extend well beyond MRI or visual macroscopic limits; therefore, removing additional brain tissue might increase survival [99]. There are recent studies backing up that SMR increases median survival without additional postoperative complications [100]. However, SMR is not always applicable. For tumors in the vicinity of eloquent areas, another concept is used, as follows:
- Maximum safe resection (MSR) consists of resecting as much brain tissue as possible without interfering with either the eloquent cortex or with the white matter tracks connected to it.
- Biopsy is where a small quantity of tissue is obtained for diagnostic rather than curative purposes.
- EOR—extent of resection (as a percentage).

The current standard of care is represented by GTR, associated with radiotherapy and chemotherapy including temozolomide [4,26,76,98]. Most studies have proven GTR as an independent predictor of increased median survival time, in comparison to NTR, STR, or partial resection.

A large study performed on 1229 patients demonstrated that patients who underwent a macroscopic resection of 100% (GTR) had a median survival time of 15.6 months, whereas patients who had an EOR between 78% and 99% had a median survival time of 9.8 months [101]. That is equivalent to a survival benefit of 5.8 months. If we put that into percentages, it means that GTR offers a 60% survival benefit compared to partial resection. Another study found that GTR offered a survival benefit of 5.4 months compared to partial resection (17.1 months comparing to 11.7) for patients undergoing radio- and chemotherapy. The same study showed that partial resection was not superior to biopsy [102]. In terms of percentages, patients undergoing GTR and optimal chemo- and radiotherapy live 46% longer than patients who only benefit from a partial resection, besides the optimal radiochemotherapy. There are several other studies that demonstrate GTR as a strong predictor of increased median survival, compared to NTR, STR, or partial resection, see Table 5 [103,104].

### 4.4. Risk of Recurrence

Proneness for recurrence is a fundamental characteristic of GBM, which represents the main reason for their poor prognosis even with the best available treatment. While virtually all GBMs will recur postoperatively, only some of them are eligible for reintervention. A study published in 2017 in World Neurosurgery proved that while only 20% of patients underwent reintervention, those who did had a significantly increased median overall survival compared to those who did not (24.2 months vs. 8.4 months). Another study analyzed 578 patients receiving up to four resections. This study showed that survival increased for each additional resection, the median survival being 6.8, 15.5, 22.4, and 26.6 months for one, two, three, or four resections respectively [105].

As far as molecular predictors of outcome for recurrent GBM are concerned, they coincide with those for primary (unoperated) GBM, the most important being IDH and MGMT promoter methylation status [106].

A large study from 2004 stratified the risks of GBM patients based on age, KPS, extent of resection, and tumor location. Low-risk GBMs were considered those arising in the frontal lobe of young patients (below the age of 40), whereas low–moderate-risk tumors were patients of the same age group with GBMs in other brain areas. Moderate–high-risk patients were those between 40 and 65 years old, with a KPS > 70, who underwent GTR or STR. Patients would be put in the high-risk group if they had one of the following factors: a KPS of 70 or lower, age above 65, and a partial resection or biopsy. This study also emphasized the fact that patients with a higher KPS are more likely to tolerate a GTR, thus improving postoperative results and survival [107].

## 5. Limitations and Future Perspectives

One limitation to the present scoping review could be the exclusion of articles whose full text is not freely available online. In present times, under the constant pressure of publishing, we believe that all major breakthroughs should be made available through open access. However, we encourage fellow scientists to send to the corresponding author published or unpublished data on the subject of the present review. We hope that the current scoping review will be the cornerstone for future study designs about the use of molecular biomarkers for the prognosis of GBM cases.

Since the introduction of temozolomide as a first-line chemotherapeutic drug for GBM, the Stupp protocol has represented the standard of care. Almost 20 years later, neither the ideal protocol nor the median survival time have improved significantly. However, a few innovative treatments have been proposed as means of increasing survival in GBM patients. For example, tumor-treating fields (TTFs) are a therapy that implies the use of intermediate-frequency (100–300 kHz) alternating electric fields to prevent cell expansion and growth by disrupting mitosis [108]. An in vitro study on mouse melanoma cells shows that intermediate-frequency electric fields interfere with cell division in the following ways: (a) prolongation of mitosis, (b) destruction of a quarter of cells by membrane rupture in the late stages of mitosis, and (c) nuclear rotation [109].

In humans, TTFs have been shown to increase overall survival in some trials. For example, in a 2017 study, Stupp et al. compared GBM postoperative patients treated with temozolomide + TTFs to patients treated with temozolomide alone. He demonstrated that patients who underwent both of the therapies had a median progression-free interval of 6 months before recurrence, compared to those solely treated with temozolomide. Moreover, patients treated with TTF and TMZ postoperatively had a median survival time of 20.9 months, compared to those only treated with temozolomide postoperatively, which had a median survival time of 16 months [110].

The tumor microenvironment (TME) of GBM includes tumoral and non-tumoral cells. The most frequent non-tumoral cells found in a GBM are the glioma-associated macrophages (GAMs). There are two main types of GAMs; IFN-γ-activated M1 macrophages, which contribute to antitumoral defense by phagocytosing GBM cells, and IL-4-activated M2 macrophages, which have an immunosuppressing activity and promote tumor progression [111,112]. Therefore, immunotherapy that targets GAMs, especially those that prevent the recruitment of the M2 subset, could represent a potential adjuvant for GBM patients [113].

The BRAFv600 mutation represents a known therapy target in other cancers, such as melanoma [114]. This mutation is frequently found in glioma, especially in epithelioid GBM [115]. In glioma, therapies that target this mutation have not been so efficient, mostly because of the impenetrability of the brain–blood barrier (BBB). There are several studies that report promising results. For instance, a series of three patients harboring malignant gliomas, two of which were glioblastomas, reported significant reduction in tumor size, increase in tumoral necrosis, and neurological improvement after the introduction of Dabrafenib, a BRAF inhibitor approved for melanoma [116].

The blood–brain barrier (BBB) is considered impenetrable for several chemotherapeutic drugs. For instance, this is a main blockage for EGFR-targeting therapies [70]. A very recent study (June 2024) has proposed ultrasound-mediated delivery of chemotherapeutic agents. This study has shown that this delivery method has increased Doxorubicine concentration in mouse brains, prolonging their overall survival [117]. We are optimistic that, as drug delivery technologies progress, so too will the outcome for patients with GBM.

Since immunotherapy has been efficient in treating other cancers, it has been hypothesized that it could at least represent an adjuvant in treating GBM. Audencel represents a vaccine containing dendritic cells loaded with autologous tumor lysate [118]. However, a 2018 randomized trial showed that Audencel increases neither progression-free survival nor overall survival [119]. This is most likely due to the highly immunosuppressive environment of GBM [118]. In terms of assessing tumor response following treatment using imaging studies, there was no difference between patients treated with the Stupp protocol and patients treated with Stupp protocol and Audencel in terms of progression-free survival, regardless of the MRI assessment criteria [120].

Another future development could be the use of AI-powered software for the integrated prognosis of GBM cases, taking into account the molecular profile, clinical aspects, and imaging data. This is already in use in other allied medical and surgical specialties such as radiology or ENT (ear–nose–throat surgery) [121].

## 6. Conclusions

As shown before, performing a gross total resection for patients with GBM undergoing optimal chemo- and radiotherapy presents a significant benefit in overall survival compared to those patients who received a subtotal or partial resection. However, this benefit ranges from 18% to 66% in most studies. On the other hand, as shown in previous sections, compared to *IDH-Wildtype* GBMs, patients with *IDH-Mutant 1/2* GBMs have an increased survival of 66% to 200%. Therefore, it is reasonable to assume that IDH status is a stronger predictor or median survival than the extent of tumor resection.

As shown before, MGMT promoter methylation status is another strong outcome predictor for patients with GBM. In the literature that we reviewed in our paper, patients with a methylated MGMT promoter lived approximately 50% to 90% longer than those with an unmethylated MGMT gene promoter. This benefit is slightly higher than the one offered by a gross total resection compared to a partial resection.

Although a strong predictor, the survival benefit presented by the methylated MGMT promoter compared to its unmethylated counterpart is lower than the benefit of having an *IDH-1 Mutant* GBM over an *IDH-Wildtype* GBM.

As far as other molecular predictors are concerned, TERT promoter mutations represent a negative outcome factor but only due to their strong association with the *IDH-Wildtype* status. They do not represent an independent factor of overall survival.

As for the EGFR, several studies have shown a tendency towards lower overall survival in GBM patients exhibiting EGFR amplification, but most of them have failed to establish a statistically significant correlation. Moreover, there are studies that have shown that high EGFR amplification can even increase the overall survival compared to low or absent amplification.

Last but not least, referring to clinical predictors of outcome, the KPS is an important predictor of survival and quality of life. Its strong correlation with QoL and survival demonstrates that we should refrain from aggressive surgery in eloquent brain areas.

As new therapies (such as TTFs) emerge, we are optimistic that the overall median survival will increase, even for *IDH-Wildtype* GBMs. For now, we can conclude that molecular profiles are stronger outcome predictors than the extent of neurosurgical resection for GBM.

## Figures and Tables

**Table 1:** Comparison of survival data reported between IDH possible scenarios.

Study	Survival *IDH-Wildtype*	Survival *IDH-Mutant* ½	Survival Benefit *IDH-Mutant* vs. *Wildtype*
Yan H. et al. (2009) [34]	15 months	30 months	15 months (100%)
Hartmann C. et al. (2010) [33]	12 months	36 months	24 months (200%)

**Table 2:** Comparison of survival data reported between unmethylated and hypermethylated MGMT scenarios for patients with GBM.

Study	Unmethylated MGMT Promoter (Abundance of MGMT Enzyme)	Hypermethylated MGMT Promoter (Low Production of MGMT Enzyme)	Median Survival Benefit
Hegi M. et al. (2005) [37]	12.2 months	18.2 months	6 months (~50%)
Weller et al. (2015) [38]	7.9 months	12.9 months	5 months (~63%)
Molenaar R. et al. (2014) [39] *	7.2 months	14.3 months	7.1 months (~98%)
Brawanski K. et al. (2023) [40] **	14 months	11 months	3 months (20%)

* This study also includes discussions about IDH-Mutant GBM, which is an entity that no longer exists according to the 2021 WHO classification. However, we have included this study as well, because it compares the benefit of having the IDH mutation to the benefit of having a hypermethylated MGMT promoter, as well as the benefit of having both beneficial mutations to the benefit of having a single beneficial mutation, which are some aspects that we consider very relevant. ** In this study, 76% of patients with unmethylated MGMT status underwent GTR (gross total resection) compared to only 60% of patients with methylated MGMT status. As we will discuss in the next sections, GTR is strongly associated with increased survival compared to partial resection. Thus, the reason that the difference in this study is so small might be the fact that patients who should theoretically have a much better outcome (methylated MGMT promoter status) underwent lower-quality surgical treatment, which diminished the differences between the 2 groups.

**Table 3:** Comparison of survival data between GBM patients with and without mutations of the TERT promoter.

Study	Median Survival Time TERT Promoter Mutation GBM Patients	Median Survival Time No TERT Promoter Mutations GBM Patients
Nonoguchi N. et al. (2013) [56]	9.3 months	9.6 months
Pekmezci M. et al. [59] *	13.2 months	18.6 months

* Not statistically significant (*p* > 0.05).

**Table 4:** Comparison of median survival given EGFR amplification.

Study	Median Survival in Months GBMs with EGFR Amplification	Median Survival in Months GBMs with Absent EGFR Amplification
Shinojima, N et al. (2003) [66]	14.4 months	20.4 months
Amirpour Z. et al. * (2020) [67]	20.6 months	27.4 months
Armocida D. et al. ** (2019) [68]	16 months	21.7 months
Hobbs J. et al. (2012) [69]	11 months (high amplification) 7.7 months (low amplification)	7.9 months

* No statistically significant difference (*p* = 0.36). ** No statistically significant difference (*p* = 0.66).

**Table 5:** Comparison of survival data reported between GTR and non-GTR scenarios.

Author	Median Survival GTR	Median Survival Non-GTR	Survival Benefit (Months)	Survival Benefit (Percentage)
Kreth F. et al. (2013) [102]	17.1 months	15.4 months	5.4	46%
Li Y. et al. (2016) [101]	15.6	9.8	5.8	60%
Jusue-Torres I. et al. (2023) [103]	20	12	8	66%
Polonara G. et al. (2023) [104]	16	14.2	1.8	12%

## Data Availability

All data are available upon request from the corresponding author.
